# Establishment of a novel three‐dimensional primary culture model for hippocampal neurogenesis

**DOI:** 10.14814/phy2.13318

**Published:** 2017-06-22

**Authors:** Tatsuya Usui, Masashi Sakurai, Hideyoshi Kawasaki, Takashi Ohama, Hideyuki Yamawaki, Koichi Sato

**Affiliations:** ^1^ Laboratory of Veterinary ToxicologyJoint Faculty of Veterinary MedicineYamaguchi UniversityYamaguchiJapan; ^2^ Laboratory of Veterinary PathologyJoint Faculty of Veterinary MedicineYamaguchi UniversityYamaguchiJapan; ^3^ Laboratory of Veterinary PharmacologyJoint Faculty of Veterinary MedicineYamaguchi UniversityYamaguchiJapan; ^4^ Laboratory of Veterinary PharmacologySchool of Veterinary MedicineKitasato UniversityTowadaJapan

**Keywords:** 3D culture, differentiation, hippocampus, neural stem cell, neurogenesis

## Abstract

New neurons are generated in the adult hippocampus throughout life and contribute to the functions of learning and memory. Nevertheless, the mechanisms by which disrupted neurogenesis regulates central nervous system (CNS) disorders are not fully understood. Here, we established a novel 3D culture system of hippocampal neurogenesis using air liquid interface (ALI) culture and Matrigel culture from mouse hippocampus tissues. After isolated mouse hippocampus tissue fragments were seeded into ALI wells and cultured in stemness‐stimulated media containing Wnt, EGF, Noggin and R‐spondin for 7 days, small spheres gradually appeared in the tissues. To identify the cell components, immunohistochemical and immunofluorescence staining were performed. Expression of a mature neuronal cell marker, NeuN was observed in the tissues just after seeding. Expression of a neural stem cell marker, Nestin was observed in the tissues at day 7. To differentiate the Nestin‐positive cells, they were passaged into Matrigel. Expression of Nestin but not an immature neuronal cell marker, doublecortin (DCX) was observed in the isolated cells. After 7 days of Matrigel culture, they showed the neurite outgrowth. Expression of Nestin was decreased compared with the one just after passaging, while DCX expression was increased. Western blotting analysis also showed Nestin expression was decreased, while expression of DCX, a neuronal cell marker, Tuj1 and a granule cell marker, Prox‐1 was increased. Here, we establish the 3D culture of hippocampus tissues that might become a novel in vitro tool for monitoring the process of hippocampal neurogenesis. Our model might shed light into the mechanisms of pathogenesis of CNS disorders.

## Introduction

The hippocampus is located under the cerebral cortex and in the medial temporal lobe of the brain. It consists of two interlocking parts, hippocampus proper and dentate gyrus (DG). Although the adult mammalian nervous system has been classically considered impossible to regenerate new neurons, recent studies suggest that the subgranular zone (SGZ) of the hippocampal DG as well as the subventricular zone (SVZ) adjacent to the lateral ventricle continuously generates new neurons throughout life (Doetsch et al. 2002) (Goncalves et al. 2016). New neurons generated at the SGZ of the DG integrate into the existing hippocampal circuitry, which plays a role in learning and formation of spatial memory (Zhao et al. 2008). Since several studies showed that neurogenesis disorders are associated with human neurological and psychiatric diseases such as epileptic seizures (Jessberger et al. 2007), Alzheimer disease (Tatebayashi et al. 2003) and schizophrenia (Hagihara et al. 2013), it is required to clarify the relationship between the process of neurogenesis, which arises from self‐renewing neural stem cells to mature neurons, and the events of these central nervous system (CNS) disorders.

Primary three‐dimensional (3D) cell culture accurately recapitulates organ structures, multilineage differentiation and physiology. The 3D epithelial organoid culture system using Matrigel was performed in the various types of gastrointestinal tissues (Sato et al. 2011). Ootani et al. established a different type of organoid culture system that mimics microenvironmental niches in the 3D culture using a collagen gel and an air‐liquid interface (ALI) method (Ootani et al. 2009) (Katano et al. 2013). In the previous study, we established human colorectal cancer tissue‐derived organoids using ALI culture, which closely resemble tumor microenvironment of the original tissues (Usui et al. 2016). Recently, a 3D culture method for generating stratified neocortical structures from human embryonic stem cells was established (Eiraku et al. 2008), (Kadoshima et al. 2013).

Primary neural stem cells have been successfully isolated from the adult rat and mouse hippocampus and used for the experiments of neuronal differentiation (Palmer et al. 1997; Bonaguidi et al. 2008). Nevertheless, the 3D primary neural stem cell culture from hippocampus tissues has not been conducted. Here, we established the culture and isolation methods of hippocampal neural stem cells expressing a neural stem cell marker, Nestin from mouse hippocampus tissues using ALI culture. The isolated neural stem cells were successfully differentiated into hippocampal neurons expressing a granule cell marker, Prox‐1 and a neuronal cell marker, MAP2ab under Matrigel culture condition.

## Materials and Methods

### Materials

To proliferate neural stem cells, mouse hippocampus tissues were cultured in the media containing stemness‐stimulating components. They were as follows: Advansed Dulbecco's Modified Eagle's Medium (DMEM) with 50% Wnt, Noggin and R‐Spondin conditioned medium; GlutaMax; 1% Penicillin‐Streptomycin (Invitrogen, Carlsbad, CA); 1 mmol/L N‐Acetyl‐L‐cysteine; 10 mmol/L Nicotinamide (Sigma‐Aldrich, St. Louis, MO); 50 ng/mL mouse EGF (PeproTech, Inc., Rocky Hill, NJ); 500 nmol/L A83‐01 (Adooq Bioscience, Irvine, CA); 3 μmol/L SB202190 (Cayman, Ann Arbor, MI). To generate the conditioned media, 1 × 10^6^ Wnt, Noggin and R‐spondin gene expressing fibroblasts were seeded in 10 mL Advansed DMEM supplemented with 10% fetal bovine serum (FBS) on 10 cm dishes. After 4 days of culture, the media were collected as a first batch and replaced them with 10 mL fresh media. After 3 days of culture, the second batch was collected and mixed with the first batch. Antibody sources were as follows: Neuronal nuclei (NeuN) (Cell Signaling Technology, Inc., Danvers, MA); Nestin (Immuno‐Biological Laboratories, Gunma, Japan); Doublecortin (DCX) (Sigma‐Aldrich); ΜAP2ab, Prox‐1 (Bioss antibodies, Woburn, MA); Tuj1 (Bethyl Laboratories, Montgomery, TX); Glial Fibrillary Acidic Protein (GFAP) (Diagnostic BioSystems, Pleasanton, CA); Valosin‐containing protein (VCP) (GeneTex, Inc., Irvine, CA). Secondary antibodies were as follows: Horseradish peroxidase **(**HRP) conjugated polymer anti‐rabbit IgG; Alexa Fluor 488 goat anti‐rabbit IgG; Alexa Fluor 568 goat anti‐rabbit IgG; (Invitrogen). HRP conjugated goat anti‐rabbit IgG (Cayman Chemical Company, Ann Arbor, MI).

## 3D culture of mouse hippocampus tissues

All studies involving mice were conducted according to the guide to animal use and care of the Yamaguchi University and approved by the ethics committee. C57BL/6 mice were obtained from Japan SLC (Hamamatsu, Japan) and mated to use 1‐week‐old mice for experiments. They were anesthetized with urethane (1.5 g/kg, i.p.) and euthanized by exsanguination. After the mice were sacrificed, their brains were immediately removed. The hippocampus tissues were isolated, put on 6 cm dish, and washed in cold HEPES buffered saline on ice. After the tissues were minced for 0.5 to 1 cm segment extensively on ice, they were washed with cold HEPES buffered saline and centrifuged at 600 g for 3 min. The pellets were embedded in a collagen gel (Nitta Gelatin Inc, Osaka, Japan) using a double‐dish culture system as previously described (Li et al. 2014) (Usui et al. 2016) and cultured for 7 days in the media.

### Isolation of neural stem cells from 3D cultured hippocampus tissues

To isolate neural stem cells from the cultured tissues, they were passaged by using a 2000 unit/mL collagenase IV (Worthington, Lakewood, NJ) as described previously (Li et al. 2014; Usui et al. 2016) and replated into new ALI collagen gels or Matrigel in 24 well plates.

### Differentiation of neural stem cells into neurons

After seeding the isolated cells into Matrigel, they were cultured for 7 days. It was reported that hippocampal neurogenesis originates from putative stem cells with a glia cell property (von Bohlen und Halbach 2011), which differentiate into neuronal progenitor cells showing a round shape. After they differentiate into immature granule cells, they start to extend their axons. Considering them, the alteration of cellular morphology was observed using a phase contrast light microscope (TE2000‐S; Nikon, Tokyo, Japan).

### Hematoxylin and eosin (H&E) staining

After cells were fixed with 4% paraformaldehyde (PFA) at 4°C overnight, they were embedded in paraffin. After deparaffinization, the 4 μm‐thick sections were stained with H&E as described previously (Usui et al. 2014). The images were obtained using a light microscope (BX‐53; Olympus, Tokyo, Japan).

### Immunohistochemical staining

After the deparaffinized sections were treated with 3% peroxidase for 15 min, they were blocked with 1% bovine serum albumin (BSA)/phosphate buffered saline (PBS) at room temperature for 1 h. They were then incubated with primary antibodies (NeuN; 1:100, Nestin; 1:100) at 4°C overnight. They were then washed three times with PBS each 5 min. After incubated with secondary antibodies (1:500) at room temperature for 1 h, they were washed three times with PBS each 5 min. They were observed using a light microscope (BX‐53).

### Immunofluorescence staining

After deparaffinization, the sections were blocked with 5% normal goat serum/PBS at room temperature for 1 h. They were then incubated with primary antibody (Nestin; 1:100, DCX; 1:200, Prox‐1; 1:200, MAP2ab; 1:200, GFAP; 1:200) at 4°C overnight. They were then washed three times with PBS each 5 min. After incubated with secondary antibodies (1:500 or 1:1000) at room temperature for 1 h, they were washed three times with PBS each 5 min. They were observed with a confocal microscope (LSM 800; ZEISS, Copenhagen, Germany).

### Western blotting

Western blotting was performed as described previously (Fujiwara et al. 2016; Kake et al. 2017). Protein lysates were obtained by homogenizing cells with Triton‐based lysis buffer (50 mmol/L Tris‐HCl (pH 8.0), 5 mmol/L EDTA, 5 mmol/L EGTA, 1% Triton X100, 1 mmol/L Na_3_VO_4_, 20 mmol/L sodium pyrophosphate, and Roche Complete protease inhibitor mixture). Protein concentration was determined using the bicinchoninic acid method (Pierce, Rockford, IL). Loading proteins (10 μg) were separated by SDS‐PAGE (10%) (80 V/0.5 A for 20 min and 120 V/0.5 A for 60 min) and transferred to a nitrocellulose membrane (25 V/0.15 A for 90 min) (Wako, Osaka, Japan). After being blocked with 0.5% skim milk, the membranes were incubated with primary antibodies (Nestin; 1:200, Prox‐1; 1:200, Tuji1; 1:200, DCX; 1:200) at 4°C overnight. And then, the membranes were incubated with secondary antibodies (1:10,000 dilution, 1 h) and ECL Pro (PerkinElmer, Freiburg, Germany). The results were visualized using a chemiluminescence analyzer equipped with a CCD camera (LAS‐3000, Fujifilm, Tokyo, Japan) and quantified using ImageJ densitometry analysis software (National Institutes of Health, Bethesda, MD). Expression level of day 0 at passage 1 represented 100% and VCP antibody was used as a loading control.

### Statistical analysis

Data are shown as mean ± SEM. Statistical evaluations were performed by using Student's *t*‐test. Values of *P *<* *0.05 were considered statistically significant.

## Results

### Three‐dimensional (3D) culture of mouse hippocampus tissues

**Figure 1:**
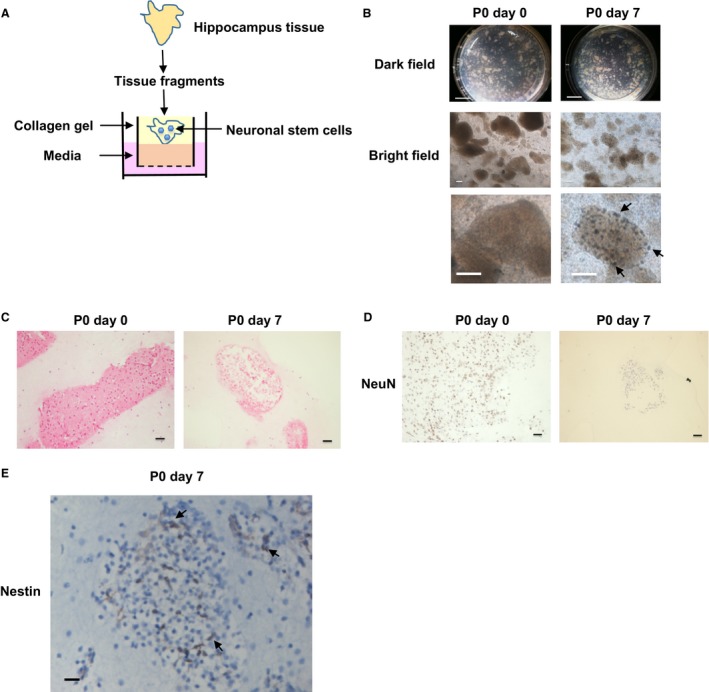
Three‐dimensional (3D) culture of mouse hippocampus tissues. Schematic experimental design of a 3D culture system. Mouse hippocampus tissue fragments were seeded in collagen gels under an air liquid interface (ALI) microenvironment and cultured in media (A). Dark and bright field pictures were taken at day 0 and 7 after seeding tissues (B). Scale bar: 5 mm (dark field), 500 μm (bright field). Enlarged pictures are shown under the original pictures. Arrows show the spheres of neural stem cells. Hematoxylin and eosin (H&E) stained sections (C) are shown. Scale bar: 100 μm. Expression of a mature neuron marker, neuronal nuclei (NeuN) (D) and a neural stem cell marker, Nestin (E) in the hippocampus tissues. Representative photomicrographs are shown (*n* = 4). Scale bar: 100 μm (D) and 50 μm (E). Arrows show the representative Nestin‐positive cells.

To recapitulate the brain structure, the 3D culture from embryonic stem cells was developed (Eiraku et al. 2008) (Kadoshima et al. 2013). However, the 3D primary culture of hippocampus tissues has not been established. We therefore cultured mouse hippocampus tissues using an ALI culture method (Fig. 1A). Isolated mouse hippocampus tissue fragments were maintained for 7 days after seeding into ALI wells (Fig. 1B). Interestingly, small spheres gradually appeared in the tissues at day 3–5 after seeding (Fig. 1B). After 7 days, we observed that the tissue structure was collapsed with decreased cell density (Fig. 1C). To confirm whether mature neuronal cells died, we performed immunohistochemical staining. Expression of a mature neuronal cell marker, NeuN was observed in the tissues at day 0, while we hardly observed it at day 7 (Fig. 1D). On the other hand, expression of a neural stem cell marker, Nestin was observed in the tissues at day 7 (Fig. 1E). These results suggest that 3D culture using ALI wells induced the death of mature neurons and increased Nestin‐positive neural stem cells.

### Isolation of neural stem cells from hippocampus tissues

**Figure 2:**
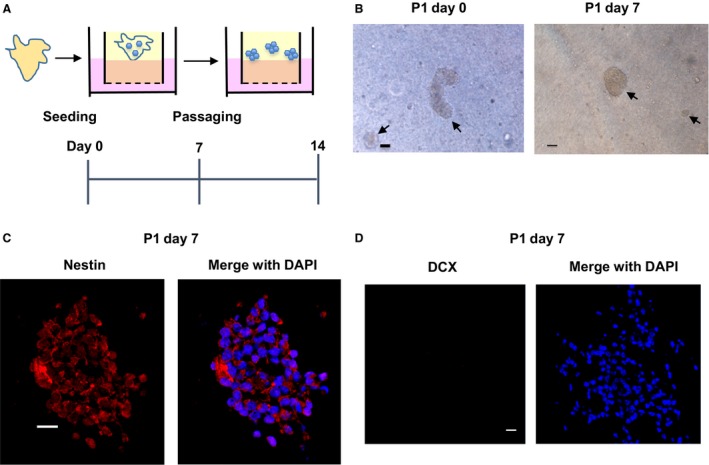
Isolation of neural stem cells from hippocampus tissues. After seeding the hippocampus fragments in collagen gels under an ALI microenvironment and cultured for 7 days, they were passaged into new ALI collagen gels using a 2000 unit/mL collagenase IV and cultured for 7 days (A). Bright field pictures were taken at day 0 and 7 post‐passage (B). Scale bar: 200 μm. Arrows show the isolated spheres. Expression of Nestin (C) and an immature neural cell marker, doublecortin (DCX) (D). Representative immunofluorescence pictures are shown (*n* = 4). Scale bar: 50 μm.

To isolate Nestin‐positive cells from the tissues, they were treated with collagenase at 37°C for 30 min and passaged into new ALI wells (Fig. 2A). After passaging, we observed the differentiated cells were removed and several numbers of spheres were formed (Fig. 2B). After 7 days, the morphology did not change compared with the one just after passaging (Fig. 2B). We next confirmed the existence of neural stem cells and immature neural cells in the spheres. DCX is a protein that promotes microtubule polymerization and exists in young neurons (Francis et al. 1999). Expression of Nestin (Fig. 2C) but not an immature neuronal cell marker, DCX (Fig. 2D) was observed in the spheres, suggesting that Nestin‐positive neural stem cells were successfully isolated by the collagenase treatment and passaging.

### Differentiation of neural stem cells into neurons in the Matrigel culture

**Figure 3:**
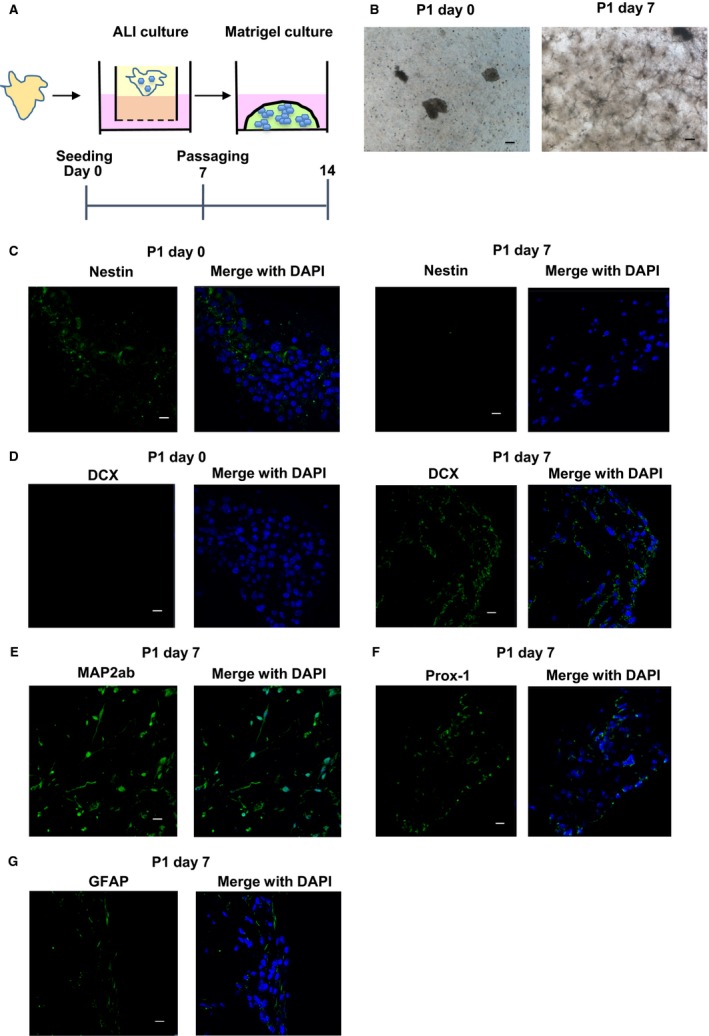
Differentiation of neural stem cells into neurons in the Matrigel culture. After seeding the hippocampus fragments in collagen gels under an ALI wells and cultured for 7 days, they were passaged into Matrigel and cultured for 7 days (A). Bright field pictures were taken at day 0 and 7 post‐passage (B). Scale bar: 200 μm. Expression of Nestin (C), DCX (D), a neuronal cell marker, MAP2ab (E), a granule cell marker, Prox‐1 (F) and an astrocytes marker, GFAP (G). Representative immunofluorescence pictures are shown (*n* = 4). Scale bar: 50 μm.

Since a recent study showed that Matrigel culture was used for differentiating embryonic stem cells into brain organoids (Kadoshima et al. 2013), we tested whether Matrigel culture mediates differentiation of the Nestin‐positive neural stem cells into mature neurons (Fig. 3A). After 7 days of Matrigel culture, the spheres showed neurite outgrowth (Fig. 3B). To identify the cell components of the differentiated cells, we performed immunofluorescence staining. Expression of Nestin was decreased at day 7 compared with the one just after passaging (Fig. 3C). On the contrary, DCX expression was increased at day 7 (Fig. 3D). MAP2ab is localized to the dendrites and found throughout the life of the neurons. (Harada et al. 2002). Prox‐1 is expressed in the hippocampal DG, which plays important roles for the maintenance of intermediate progenitors during adult neurogenesis (Lavado et al. 2010). Prox‐1 is also required for the maturation of the granule cells. We next confirmed that expression of a neuronal cell marker, MAP2ab (Fig. 3E) and a granule cell marker, Prox‐1 (Fig. 3F) was observed in the differentiated cells. Since astrocytes are known to promote hippocampal neurogenesis (Song et al. 2002), we investigated whether astrocytes were included in the Matrigel culture using astrocytes marker, GFAP. In the Matrigel culture, GFAP‐positive cells were observed (Fig. 3G).

### Expression changes of neuronal differentiation marker proteins in the Matrigel culture

**Figure 4:**
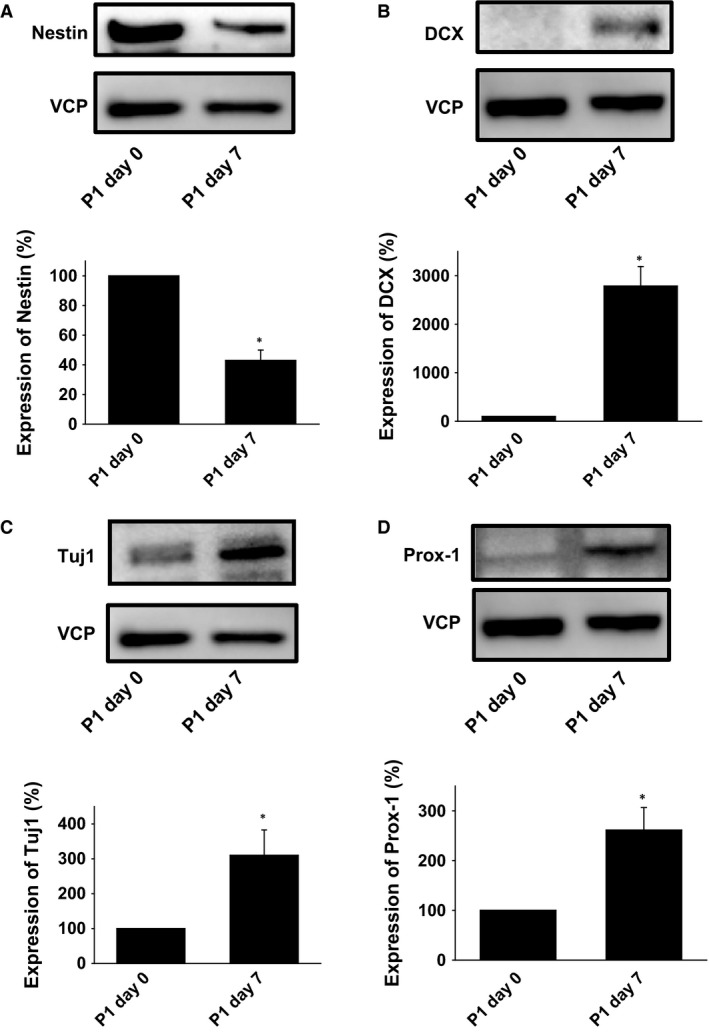
Expression changes of neuronal differentiation marker proteins in the Matrigel culture. After the hippocampus tissues were cultured in ALI wells and seeded into Matrigel, expression of Nestin (A, *n* = 4), DCX (B, *n* = 5), a neuronal cell marker, Tuj1 (C, *n* = 3) and Prox‐1 (D, *n* = 4) was determined by Western blotting at day 0 and 7 post‐passaging. Equal protein loading was confirmed using total VCP antibody. *P <* *0.05 versus P1 Day 0.

We finally checked the expression of neuronal differentiation marker proteins using Western blotting. Tuj1 is a neuron‐specific marker for newly generated cells (Parent et al. 1997). Corresponding with the data of immunofluorescence staining, Nestin expression was significantly decreased at day 7 (Fig. 4A). Expression of DCX (Fig. 4B), Tuj1 (Fig. 4C) and Prox‐1 (Fig. 4D) was significantly increased at day 7.

## Discussion

**Figure 5:**
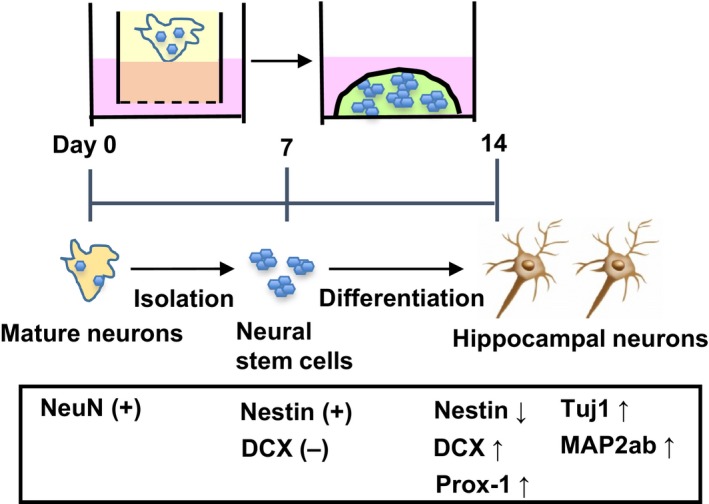
Summary of the present results. Neural stem cells were isolated using ALI culture and passaging. They were differentiated into hippocampal neurons in the Matrigel culture, expressing several neuronal cell markers and a granule cell marker.

In the present study, we for the first time demonstrated the novel method of isolation and differentiation of primary mouse neural stem cells using two types of 3D culture. The major findings of the present study are as follows (Fig. 5): (1) ALI culture of mouse hippocampus tissues decreased mature neurons expressing NeuN and increased neural stem cells expressing Nestin, (2) after passaging, neural stem cells were successfully isolated as the spheres, (3) Matrigel culture mediates differentiation of the isolated neural stem cells into hippocampal neuronal cells expressing DCX, Tuj1, Prox‐1 and MAP2ab. Collectively, our results indicate that these methods are useful as the novel tools to investigate the process of primary neuronal differentiation in the 3D culture.

Previously, mouse neural stem and precursor cells were isolated according to the following method (Bull and Bartlett 2005): hippocampus tissues were minced and enzymatically digested with 0.1% trypsin‐EDTA. After centrifugation, the cells were filtered and seeded on the 2D culture plate in the media containing the growth factors such as epidermal growth factor (EGF) and fibroblast growth factors (FGF)‐2. It was also shown that Noggin is required for the maintenance of neuronal stem cells (Bonaguidi et al. 2008). In the present study, we cultured hippocampus tissues using ALI culture method and successfully isolated many Nestin‐positive cells in the media containing EGF and Noggin, which could differentiate into hippocampal neurons. We also showed that differentiated neural cells were surrounded by astrocytes in the Matrigel culture (Fig. 3G). Since it was reported that neural stem and progenitor cells are regulated by microenvironmental niches such as astrocytes (Hagg 2005) (Ma et al. 2005), which are essential for hippocampal neurogenesis (Song et al. 2002), we speculate that ALI and Matrigel culture might maintain the microenvironmental niches in the hippocampus, which is necessary for growing neural stem cells that enable the hippocampal neurons.

In the present study, we demonstrated that 3D cultured neural stem cells differentiate into neurons expressing Prox1, Tuj1 and DCX (Figs 3 and 4). It was reported that brain derived neurotrophic factor (BDNF), which promotes neurogenesis, is necessary for neuronal progenitor cells to differentiate (Yu et al. 2014). In the same report, coculture of hippocampus astrocytes was also needed for the differentiation. In the present study, we added Wnt, Noggin, EGF and R‐spondin but not BDNF to the culture media. Since we observed the isolated neural stem cells contained an astrocyte marker, GFAP‐positive cells (Fig. 3G), we suppose that some neural stem cells differentiate into astrocytes, which secret growth factors to help differentiation into neurons.

Disorders of hippocampal neurogenesis have been implicated in a variety of the pathogenesis of neurological diseases (Jun et al. 2012). In epileptic seizure model rats, the abnormal dendrites emerge in the granule neurons. Normal granule neurons extend dendrites toward the molecular layer, while abnormal ones extend toward the hilus (Jessberger et al. 2007). Hippocampal neurogenesis deficits are also linked to cognitive defects such as depression (Patricio et al. 2013), Alzheimer's disease (Demars et al. 2010), bipolar disorder (Valvezan and Klein 2012), and schizophrenia (SCZD) (Tamminga et al. 2010). In SCZD patients, the number of immature neuronal stem cells increased in the DG (Yamasaki et al. 2008). Nevertheless, the detailed mechanisms of these diseases are not fully understood because the examining postmortem tissues can only provide information at the end point of the disease. Therefore, using our mouse primary 3D hippocampal neurogenesis model might shed light to the mechanisms of the disorders of hippocampal neurogenesis and identify potential targets for the diseases.

In summary, we for the first time established the 3D culture system of primary DG granule neural stem cells from hippocampus tissues. They could recapitulate the marker expression profile during the process of hippocampal neurogenesis. This model thus might shed light into the mechanisms of pathogenesis of CNS disorders. Further studies on 3D hippocampus tissue culture contribute to develop a promising tool for the drug screening against CNS disorders.

## Conflict of Interest

None declared.
